# Inborn Errors of Immunity With Immune Dysregulation: From Bench to Bedside

**DOI:** 10.3389/fped.2019.00353

**Published:** 2019-08-27

**Authors:** Ottavia Maria Delmonte, Riccardo Castagnoli, Enrica Calzoni, Luigi Daniele Notarangelo

**Affiliations:** ^1^Laboratory of Clinical Immunology and Microbiology, Division of Intramural Research, National Institute of Allergy and Infectious Diseases, National Institutes of Health, Bethesda, MD, United States; ^2^Foundation IRCCS Policlinico San Matteo, Department of Pediatrics, University of Pavia, Pavia, Italy; ^3^Department of Molecular and Translational Medicine, A. Nocivelli Institute for Molecular Medicine, University of Brescia, Brescia, Italy

**Keywords:** primary immunodeficiency diseases (PID), immunedysregulation, combined immunodeficiency, precision medicine, small molecules, monoclonals, gain of function (GOF)

## Abstract

Inborn errors of immunity are genetic disorders with broad clinical manifestations, ranging from increased susceptibility to infections to significant immune dysregulation, often leading to multiple autoimmune phenomena, lymphoproliferation, and malignancy. The treatment is challenging as it requires careful balancing of immunosuppression in subjects at increased risk of infections. Recently, the improved ability to define inborn errors of immunity pathophysiology at the molecular level has set the basis for the development of targeted therapeutic interventions. Such a “precision medicine” approach is mainly bases on the use of available small molecules and biologics to target a specific cell function. In this article, we summarize the clinical and laboratory features of various recently described inborn errors of immunity associated with immune dysregulation and hyperinflammation in which mechanism-based therapeutic approaches have been implemented.

## Introduction

With the increased availability of high-throughput DNA sequencing, the number of genes associated with inborn errors of immunity [historically named primary immune deficiency disorders (PIDs)] has exponentially increased over the last decade. The most recent PID classification from the International Union of Immunological Sciences includes more than 350 genes, and ~50 of these have been discovered in the last 2 years (1). In addition to the identification of novel PID-associated genes, it has been recognized that distinct clinical phenotypes may be sustained by Gain of Function (GOF) or Loss of Function (LOF) mutations in the same gene. Finally, various degrees of activity of mutant proteins due to hypomorphic and hypermorphic mutations may also cause PID phenotypic variability (1, 2).

The clinical features of PIDs are broad, ranging from increased susceptibility to infections to significant immune dysregulation, often leading to multiple autoimmune phenomena, including cytopenias and solid organ autoimmunity, in addition to lymphoproliferation and malignancy. The treatment of immune disorders with coexisting immune deficiency and immune dysregulation is challenging, as it requires careful balancing of immunosuppression in subjects at increased risk of infections. In most recent years, the growing ability to define PID pathophysiology at the molecular level has set the basis for the development of targeted therapeutic interventions. New drugs have been developed or repurposed to modulate intracellular pathways whose function is increased or diminished as a result of a specific genetic defect (Table 1). Such a “precision medicine” approach often permits to selectively target a specific cell function instead of broadly affecting the entire immune system, and may even permit to avoid deleterious side effects on other tissues. In this manuscript, we summarize the laboratory and clinical features of various recently described PIDs, focusing in particular on disorders associated with immune dysregulation in which targeted therapeutic approaches have been implemented according to the recent knowledge of the molecular mechanisms underpinning these diseases and the most common clinical manifestations.

**Table 1:** Targeted therapies used in disorders of immunedysregulation and hyperinflammation.

**Molecular Target**	**Molecular Structure**	**Drug**	**Indication**
CD52	mAb	Alemtuzumab	Hemophagocytic lymphohistiocytosis
JAK	Small molecule inhibitor	Ruxolitinib	
IFN-γ	mAb	Emapalumab	
mTOR	Macrolide compound	Sirolimus	NLCR4-GOF POMP deficiency CTLA-4 haploinsufficiency APDS
B7-1 (CD80) B7-2 (CD86)	CTLA-4 IgG fusion protein	Abatacept	CTLA-4 haploinsufficiency LRBA deficiency
		Belatacept	CTLA-4 haploinsufficiency
IL-1R	Recombinant human IL-1R antagonist	Anakinra	Cryopyrin-associated periodic fever syndromes
IL-1β	Antihuman IL-1 IgG1 mAb	Canakinumab	CAPS FCAS MWS DIRA
	IgG1 linked to IL-1R and IL-1R accessory protein	Rilonacept	
IL-6R	IgG1κ recombinant humanized mAb	Tocilizumab	STAT3-GOF
TNF-α	Fusion protein	Etanercept	SAVI CANDLE syndrome POMP deficiency
	Chimeric mAb	Infliximab Adalimumab	
	Humanized mAb		
JAK1 and JAK 2	Small molecule inhibitor	Ruxolitinib	STAT3-GOF* STAT1-GOF CANDLE syndrome
JAK 1 and JAK3		Baricitinib Tofacitinib	

P110δ		Leniolisib	APDS
IL-18 binding protein	Recombinant IL-18 binding protein	Tadekinig-α	NLCR4-GOF
B-lymphocyte stimulator	Human mAb IgG1-λ	Belimumab	Autoimmune cytopenias
Plasma cells	Proteasome inhibitor	Bortezomib	
C5	Recombinant IgG2/4κ	Eculizumab	
CD22	Humanized mAb	Epratuzumab	
Bruton's tyrosine kinase	Small molecule	Ibrutinib	
CD20	Human/murine IgG1κ mAb	Rituximab	
CD38	Human mAb	Daratumumab	

* Only Ruxolitinib and Tofacitinib.

*JAK, Janus Kinase; CTLA-4, Cytotoxic T lymphocyte antigen-4; IL-1R, Interleukin-1 receptor; IL-1, Interleukin-1; mAb, monoclonal antibody; IL-18, Interleukin-18; mTOR, mammalian target of rapamycin; IL-1β, Interleukin-1 beta; IL-6R, Interleukin-6 receptor; TNF-α, Tumor necrosis factor alpha; JAK1, Janus Kinase 1; JAK 2, Janus Kinase 2; JAK3, Janus Kinase 3; PI3Kδ, Phosphoinositide 3-kinase delta; NLRC4-GOF, NLR Family CARD Domain Containing 4; POMP, Proteasome maturation protein; APDS, activated PI3K-delta syndrome; LRBA, lipopolysaccharide (LPS)-responsive and beige-like anchor protein; DIRA, Deficiency of Il-1 Receptor Antagonist (Il-1 RA); STAT3-GOF Signal transducer and activator of transcription 3 gain of function; SAVI, STING-associated vasculopathy with onset in infancy; CANDLE, chronic atypical neutrophilic dermatosis with lipodystrophy and elevated temperature; STAT1-GOF, Signal transducer and activator of transcription 1 gain of function*.

## Precision Medicine in Disorders of Immunedysregulation

### CTLA4 Haploinsufficiency

Cytotoxic lymphocyte antigen 4 (CTLA4, CD152), is a receptor on T cells that inhibits cell activation and immune response. CTLA4 binds to 2 different ligands on antigen presenting cells (APC), CD80 and CD86 (3). Upon ligand binding, CTLA4 produces an inhibitory signal to limit T cell activation and proliferation. This is the opposite of what happens when CD80/86 bind to CD28, an T cell co-stimulatory molecule (3) (**Figure 2B**). CTLA4 expression is crucial for T regulatory cell function and immune-tolerance as well. Identification of CTLA4 as a key immune regulator has led to the production of a fusion molecule composed of the extracellular domain of CTLA4 joined to the Fc region of IgG1 (abatacept and belatacept) that is able to inhibit T cell activation *in vivo* (4) (**Figure 2B**). Side effects of these drugs depend on their immune suppressive activity that results in increased susceptibility to infections (especially viral) and malignancy. CTLA4 haploinsufficiency is due to heterozygous germline mutations in the *CTLA4* gene. Two groups originally reported the presence of *CTLA4* mutations in immunodeficient individuals affected by viral and sinopulmonary infections, associated with autoimmunity and lymphoproliferation (5, 6). Clinical and laboratory findings were consistent with common variable immunodeficiency (CVID) but patients also suffered from significant autoimmune cytopenia, along with T cell infiltrates in the lungs, gastrointestinal tract, bone marrow, and nervous system. Functional studies showed hyperactivation of effector T cells; moreover, FOXP3+ Treg cells had diminished CTLA4 expression and displayed impaired suppressor function (6). In addition, patients had decreased CTLA4 expression on the surface of activated conventional T cells, suggesting that impaired expression of this molecule may cause both defective capacity to extinguish T cell responses and to control self-reactive T cells that have not been deleted in the thymus. Furthermore, CTLA4 haploinsufficient patients have a progressive reduction of B cells with increased proportion of autoreactive CD21^low^ B cells (5). Importantly, the disease is characterized by incomplete penetrance and variable expressivity (5, 6). More recently, a cohort of 133 patients with CTLA4 has been described by Schwab et al. broadening the clinical and immunological spectrum associated with this disease (7). Clinical manifestations in this series included respiratory and gastrointestinal disease, non-malignant lymphoproliferation, severe or refractory autoimmune cytopenias. Pulmonary findings included multiple upper and lower respiratory tract infections, bronchiectasis, lymphocytic interstitial lung disease, and lung fibrosis. Gastrointestinal manifestations were present, with enteropathy and Crohn's-like colitis being often particularly severe. The immunological phenotype included variable degrees of hypogammaglobulinemia and impaired response to immunizations, low numbers of CD4 T-cell, and B-cell defects of maturation (7). Initially, patients with CTLA4 haploinsufficiency were treated only with rapamycin to decrease T cells hyperactivity, but abatacept and belatacept have shown to be an effective targeted treatment to control the immune dysregulation of this disorder (4).

The first CTLA4 patient successfully treated with Abatacept was a 14-year-old girl affected by severe enteropathy and chronic diarrhea, autoimmune cytopenia, and autoimmune hepatitis. Therapy with abatacept improved the diarrhea, the autoimmune hemolytic anemia and avoided the use of other immunosuppressant medication (8). In the cohort described by Schwab et al. eleven patients received abatacept or belatacept with amelioration of lymphoproliferation in the lungs, lymphadenopathy, autoimmune thrombocytopenia, and colitis.

In the same cohort, sirolimus was administered to 13 patients with clinical improvement (reduced splenomegaly, lymphadenopathy, and cytopenia) (7).

Navarini et al. described a case of CTLA4 haploinsufficiency with refractory autoimmune enterocolitis, that improved significantly after treatment with vedolizumab, a humanized monoclonal antibody that targets T cells expressing the gut homing receptor, α4β7 integrin (9). However, vedolizumab did not reverse the hypogammaglobulinemia and pure red cell aplasia that were also present in the same patient (9).

The use of abatacept and belatacept in CTLA4 deficiency seems very promising, especially as first line therapy to control manifestations of immune dysregulation; however, the increased susceptibility to infections that the patients may develop during treatment may be a challenge in the context of lifelong therapy. For this reason, Hematopoietic stem cell transplantation (HSCT) should be carefully considered as a possible definitive therapy in patients with CTLA4 haploinsufficiency. Results of HSCT are limited to a small cohort of patients, but have been encouraging, supporting the idea that this may represent an optimal drug to utilize in patients with severe disease manifestations that experience viral reactivations or with only partial improvement after therapy with immunomodulatory drugs (10).

### LRBA Deficiency

Lipopolysaccharide-responsive and beige-like anchor (LRBA) is a cytosolic protein that co-localizes with CTLA4 in recycling endosomes; when LRBA is missing, the CTLA4 protein is targeted to lysosomal degradation and its expression on Treg cells and activated conventional T cells is significantly decreased (11). Deficiency of LRBA leads to an autosomal recessive form of CID. Patients present early in life with infections, autoimmunity and hypogammaglobulinemia (12). Since the original description, the clinical phenotype of the disease has broadened to include many more conditions of immune dysregulation like enteropathy, autoimmune hemolytic anemia and idiopathic thrombocytopenia. Autoimmune liver disease, diabetes type I, myasthenia gravis, hyper/hypothyroidism, uveitis, alopecia, polyarthritis, and gastric adenocarcinoma have been also reported (12–16). Non-malignant lymphoproliferation leading to splenomegaly and lymphadenopathy, and respiratory complications including interstitial lung disease, granulomas, and bronchiectasis mainly related to viral and bacterial infections, are also common (14, 16). Central nervous system (CNS) inflammation has been observed in one fourth of the affected individuals, and may manifest with demyelination, but also brain atrophy and granulomas (14).

Some patients may “present with enteropathy and polyendocrinopathy, a phenotype that mimics the immune dysregulation, polyendocrinopathy, X-linked (IPEX) syndrome” (17). The immunological phenotype of LRBA deficiency is characterized by low IgG, and decreased proportion of switched memory B cells (12, 14, 16). The presence of an increased proportion of double negative T cells has been also reported (18). Most of the patients present with reduced numbers and impaired function of Treg cells (17), that express decreased levels of FOXP3, CD25, and CTLA4 proteins (11, 17).

The functional interplay of LRBA and CTLA4 proteins may explain the phenotypical similarities among the two diseases, and has offered a rational basis to attempt treatment with abatacept also for LRBA deficiency (11). Use of this treatment in 3 patients has shown that abatacept may be effective in reverting lymphocytic interstitial lung disease and cytopenias; however, the enteropathy was not as responsive and required addition of sirolimus and other immunosuppressant drugs. Moreover, treatment with abatacept was associated with improvement of the immunological phenotype, as shown by increased numbers of naïve T- cells, and partially restored vaccine response after immunization with polysaccharide antigens. Of note several years of treatment did not lead to increased rate of autoimmune or infectious complications (11).

In patients with LRBA deficiency, *in vitro* studies have demonstrated that chloroquine, an inhibitor of lysosomal degradation, may also reverse CTLA4 expression loss (11), suggesting that both chloroquine and hydroxychloroquine maybe used as immunomodulatory drugs in this disease. Like in CTLA4, also in LRBA deficiency, HSCT is the only potentially curative treatment; however, only few patients have been transplanted so far and additional data are needed to assess safety and efficacy (16, 19).

### Activated Phosphoinositide 3-Kinase δ Syndrome

Activated phosphoinositide 3-kinase δ syndrome (APDS) is a combined immunodeficiency (CID) disorder due to gain-of-function (GOF) mutations in either the *PIK3CD* or the *PIK3R1* genes. These genes encode for the p110 δ catalytic subunit and the p85α regulatory subunit of phosphoinositide 3-kinase (PI3K), respectively. PI3K phosphorylates phosphatidylinositol-4,5 biphosphate (PIP2) to phosphatidylinositol-3,4,5 trisphosphate (PIP3), an important mediator of PI3K downstream cellular pathways including mTOR. In 2014 two different groups in United States (20) and Europe (21) reported that heterozygous GOF mutations in *PIK3CD* lead to a CID phenotype (APDS1). Soon after it was reported that heterozygous LOF mutations in PIK3R1 result in a similar clinical phenotype (APDS2) (22). APDS1 is caused by different heterozygous mutations but the E1021K amino acid substitution is by far the most common. The *PIK3R1* gene most common mutation leading to APDS2 is a heterozygous donor splice site mutation causing skipping of exon 11 (22–24). The consequent loss of p110δ-binding site in exon 11 results in loss of p85α subunit-mediated inhibitory control on p110δ, and consequently causes hyperactivation of the PI3K pathway (22). The clinical phenotypes of APDS1 and APDS2 significantly overlap (25, 26). Both diseases are characterized by T cells senescence, immunodeficiency, lymphoproliferation, autoimmunity, and lymphoma. The onset of the disease is typically in childhood with sino-pulmonary infections that are most commonly due to *Streptococcus pneumoniae* and *Haemophilus influenzae* and often lead to bronchiectasis over time (25). Recurrent or persistent infections due to *herpesviridae*, such as EBV, cytomegalovirus, HSV and VZV are also frequent (25). Lymphoproliferation manifesting as splenomegaly and/or hepatomegaly and lymphadenopathy, is present in most of the affected individuals, and autoimmunity, lymphoid hyperplasia of the airways and gut, developmental delay and enteropathy are also common (27). APDS patients are at higher risk of lymphomas (particularly EBV-driven B cell lymphoma) (25, 26).

Growth delay has been documented in around half of APDS2 patients, but it is not a clinical feature of APDS1. This may be due to dysregulated activity of p110α and p110β PI3K subunits (23, 24, 26). The immunological phenotype of APDS includes both T cell abnormalities with decreased naïve T cells, increased T effector memory cells and exhausted T_EMRA_ cells and increased T follicular helper (Tfh) cells. Patients also have B cell impairment as indicated by variable degrees of hypogammaglobulinemia, elevated IgM levels and higher proportions of transitional B cells, reduced numbers of switched memory B cells and diminished response to immunization (20). Functional testing on T and B cells in APDS patients demonstrated increased AKT and S6 phosphorylation as a result of augmented mTOR signaling (20), thereby supporting the use of mTOR-targeted therapy to control the disease (28). Treatment of APDS includes antimicrobial prophylaxis and immunoglobulin replacement to prevent infectious complication (26). Multiple immune suppressive regimens have been attempted to control lymphoproliferation and autoimmunity; the best results have been obtained with mTOR inhibitors (such as rapamycin) and rituximab (28). Hematopoietic stem cell transplantation (HSCT) showed successful reversion of the clinical phenotype, however graft failure, and viral reactivation post-transplant have been frequently observed (29, 30).

Characterization of the molecular mechanisms underpinning APDS prompted use of selective PI3K δ inhibitors as a possible therapeutic option. Two phase 2 trials are currently open to establish safety and efficacy of these drugs. The first trial is based on oral administration of leniolisib (NCT02435173), while the other one is based on inhaled nemiralisib (NCT02593539). Dose-escalating administration of leniolisib over a period of 12 weeks was safe and effective in decreasing lymphadenopathy and splenomegaly. Cytopenias also improved toward the end of the treatment (31).

Even tough targeted therapy with PI3Kδ inhibitors and mTOR inhibitors has shown to be useful in patients with APDS, there are still patients with this disease that do not respond to targeted treatments or that may develop significant side effects from them. These specific groups of patients will require alternative treatment, including HSCT. Moreover, the long-term safety profile of PI3Kδ and mTOR inhibitors in patients with APDS has not been completely characterized, especially considering some evidence that P110δ inhibitors may increase genomic instability in B cells trough an AID-driven mechanism (32). Furthermore, it is important to consider that therapy with rapamycin does not decrease lymphoma risk (28).

### STAT1 and STAT3 GOF

**Figure 1:**
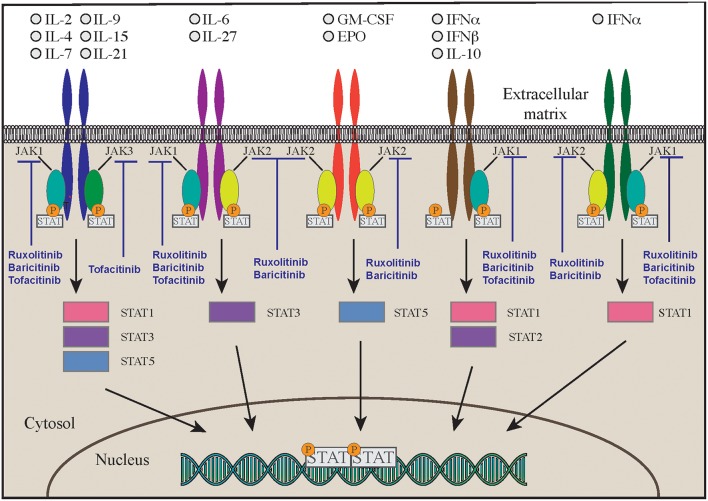
JAK-STAT signaling inhibition by multiple Jak inhibitors.

Signal Transducers and Activator of Transcription (STAT) molecules include seven different proteins that are expressed by immune and non-immune cells and are crucial in regulating immune and inflammatory responses. After the binding of different cytokines and growth factors—type I, type II, type III interferons, IL-6, EGF, PDGF, IL-21, IL-23- to their own receptor on the cell surface, four different Janus kinases are recruited intracellularly to the receptor intracytoplasmic tail. Upon activation and phosphorylation, JAKs phosphorylate in turn the intracellular tail of the cytokine receptor, offering a binding site for the SH2 domain of cytosolic STAT molecules that, upon phosphorylation, form heterodimers or homodimers, and translocate to the nucleus to promote transcription of target genes that regulate various immune pathways and control cell proliferation, differentiation, survival, and death (33) (Figure 1). Although JAK and STAT protein functions overlap in the transduction of the signaling mediated by cytokine, nonetheless mutations in individual *JAK* and *STAT* genes are associated with distinctive features, with some degree of similarity (33).

In particular, STAT1 is activated upon binding of type I and type II interferons, IL-2, IL-21, IL-10 and other cytokines to their cognate receptors. The signal is transmitted through JAK1, JAK2, and JAK3 in different combinations (34). In 2003 it was first described that biallelic LOF mutations of *STAT1* lead to severe susceptibility to mycobacterial and viral infections due to impaired response to both type I and type II interferons (35). It was subsequently shown that heterozygous mutations with dominant negative effect cause susceptibility to mycobacterial disease (36). Furthermore, heterozygous GOF mutations in *STAT1* lead to chronic mucocutaneous candidiasis (CMC) (37, 38). The identification of many more patients with STAT1 GOF mutations allowed to broaden the phenotypical spectrum of the disease. Toubiana et al. reported a cohort of 274 patients collected from multiple centers. Virtually all subjects suffered from CMC in childhood (39), and 74% of them suffered also from bacterial infections of the skin and respiratory tract, mostly due to *Staphylococcus aureus*. Viral infections, especially due to *Herpesviridae*, and mycobacterial disease were also reported (39). Severe and invasive fungal infection were due not only to Candida but included mucormycosis (40), coccidioidomycosis, histoplasmosis, and aspergillosis (41). Immune dysregulation and autoimmunity are a common feature of the disease and are present in 30% of the patients (42). Among the most common autoimmunity there are hypothyroidism, type 1 diabetes, autoimmune cytopenias, and systemic lupus erythematosus however also enteropathy, arthritis, and multiple sclerosis have been documented (39). Cerebral aneurysms and vasculopathy are frequently observed and pose patients with STAT1 GOF mutations at risk of intracranial bleed if not timely diagnosed (39, 42). Malignancies (including squamous cell carcinoma) may also occur (39).

Most of the mutations fall in the DNA-binding and coiled-coil domains of the protein (37, 39) leading to increased STAT1 expression, increased STAT1 phosphorylation or delayed dephosphorylation after stimulation with IFN-α, IFN-γ, IL-27 (42, 43). A variable degree of immunodeficiency has been described in patients with STAT1 GOF mutations, including decreased number and function of T, B, and/or NK cells, and hypogammaglobulinemia (39). Furthermore, patients have decreased proportion of memory B cells, and circulating Tfh cells have an aberrant phenotype, with decreased numbers of CCR6+ cells (effective B-helper Tfh cells), and increased expression of IFN-γ and of programmed death 1 (PD1) proteins (44). The Immune dysregulation and autoimmunity of the disease may be refractory to conventional treatment and challenging to treat. HSCT is in the only curative treatment; however, low survival and increased rates of graft failure have been reported after HSCT (45). Medical therapy is based on long-term use systemic anti-fungal agents that may not always be effective due to the onset of resistance to azole. Anti-bacterial and anti-viral medications, and use of immunosuppressive drugs are often needed as well. Despite these therapies, the mortality rate by 60 years of age is more than 10% in patients without invasive infections, malignancy, and/or aneurysms, but is around 70% in those with multiple autoimmunity. The molecular understanding of Janus kinase-STAT pathway offered the opportunity to use targeted pharmacologic inhibitors in patients with STAT1-GOF (46–48). Currently 5 different small molecule Jak inhibitors (Jakinibs) are available: tofacitinib (JAK1 and JAK3 inhibitor), ruxolitinib (JAK1 and JAK2 inhibitor), baricitinib (JAK1 and JAK2 inhibitor), filgotinib (a more selective JAK1 inhibitor), and decernotinib (a selective JAK3 inhibitor) (49, 50) (Figure 1). The choice of which Jakinib to use can be driven by the different toxicity profiles, availability of the formulations, and disease to treat.

Recently Forbes et al. reported 11 subjects affected by STAT1 GOF treated with a Jakinib. Clinical features included: severe fungal infections in six patients (including CMC in 5 patients and disseminated coccidiomycosis in 1 patient), cytopenias in 6 patients, autoimmune enteropathy (mostly TPN dependent) in 5, and hepatitis in 5 other patients. Polyendocrinopathy and interstitial lung disease were also present in some patients (46). Therapy with Jakinibs led to improvement of cytopenias, interstitial lung disease, and TPN-dependent enteropathy. Patients also improved their ability to control infections to a certain extent. CMC was responsive to the treatment (46) while the disseminated coccidiomycosis progressively worsened and the patient eventually died. Another case was described in the same cohort with severe fungal infection and unfavorable outcome despite therapy with a Jakinib, pointing out that such treatment may be insufficient to reverse systemic fungal infection (46). Treatment with baricitinib was able to reduce the interferon signature and downstream interferon activation in one patient (48). Treatment with Jakinibs *in vitro* leads to reduction in STAT1 phosphorylation and improved NK-cell cytotoxicity in peripheral blood mononuclear cells from affected patients (51, 52). Furthermore, normalization of the proportion of TH1, TH17, and Tfh cells has been associated with clinical improvement in patients undergoing treatment (47). Therapy with Jakinibs may cause elevation in liver enzymes and mild decrease in the platelet count. EBV, CMV, BK, JC viremia should always be monitored in patients on treatment, especially if more than one immunosuppressive agent is administered in combination with Jakinibs. Herpes zoster reactivation have been described in two patients receiving this treatment, and prophylaxis with acyclovir o valacyclovir needs to be considered (46). HSCT has been attempted in patients with complicated disease, with controversial results (39, 45, 53).

STAT3 is activated upon intracellular signaling from type I, II, and III interferons, IL-6, IL10, and IL-21. The original description of heterozygous STAT3 GOF mutations included patients with early-onset autoimmunity or type 1 diabetes in infancy (54). Experiments based on a luciferase reporter assay have shown that HEK293T cells transfected with a mutant STAT3 had increased transcriptional activity as compared to cells transfected with wild-type STAT3 (54). Moreover, patients displayed decreased Treg cells and CD4+ T cells that were mainly skewed to the TH1 phenotype (54). More patients with *STAT3* GOF mutations were identified shortly thereafter (55), expanding the clinical phenotype to non-malignant lymphoproliferation including lymphadenopathy, splenomegaly, and interstitial pneumonia and recurrent infections due to non-tuberculous mycobacteria, fungi, and viruses. Acquired short stature is also a peculiar feature of the disease (55–57). Enteropathy and cytopenias were the most common autoimmune manifestations (55). Attentive studies of these families revealed that there are some genetically affected members with absent or very mild phenotype consistent with incomplete penetrance and variable expressivity (58). The immunological characteristics of subject affected by *STAT3* GOF germline mutations showed that these patients may suffer from T cell lymphopenia together with elevated proportion of double negative TCRαβ+ T cells, hypogammaglobulinemia with terminal B cell maturation arrest and a reduced number of circulating dendritic cells, eosinophils, TH17 cells, and natural killer cells (55, 58). All of these immunological abnormalities are caused by enhanced transcriptional activity of STAT3 or delayed kinetics of STAT3 dephosphorylation. Furthermore, *STAT3* GOF mutations lead to decrease in STAT1 and STAT5 phosphorylation (58). Decreased levels of phosphorylated STAT5 have been proposed as a possible mechanism leading to diminished growth and stature post-natally, reflecting impaired signaling through the growth hormone receptor (59). The severe immune dysregulation described in subjects with *STAT3* GOF mutations is linked to the well-documented role of STAT3 signaling in promoting inflammation and TH17 cell differentiation, and suppressing Treg cells function (60, 61). Characterization of the molecular abnormalities underlying the disease offered the basis to treat these patient with an IL-6-targeted therapy. Therapy with tocilizumab (an anti-IL6R monoclonal antibody) led to significant improvement of contractures, inflammatory markers, and normalizations of the proportion of TH17 cells in one patient with *STAT3* GOF mutation who suffered from severe arthritis and scleroderma-like disease that were refractory did to conventional immunosuppressant therapies (58). Three additional patients did benefit from tocilizumab administration to control autoimmune hepatitis, lymphoproliferation, enteropathy, and interstitial lung disease. Unfortunately, this treatment was not sufficient to completely reverse the immune dysregulation, and Jakinibs had to be added as well. In 3 other patients, tocilizumab and a Jakinib were initiated at the same time, leading to successful regression of manifestations of immune dysregulation. These results support the idea that the combination of IL-6 inhibitors and Jakinib therapy is an effective therapeutic option, and both agents should be considered as combination therapy in the treatment of immune dysregulation in patients with *STAT3* GOF mutations (46). Finally, two patients with refractory autoimmunity underwent HSCT. The first patient died due to disseminated adenovirus infection post-transplant; the second was reported to be alive and disease-free (58).

### Precision Medicine in Autoimmune Cytopenias

Autoimmune cytopenias, like immune thrombocytopenic purpura (ITP), autoimmune hemolytic anemia (AIHA), and autoimmune neutropenia (AN) are among the most common manifestations of immune dysregulation in patients with PID. Two different studies in 2005 and 2011 reported that “a diagnosis of PID was achieved in 13% of children with AIHA and up to 50% of children with Evans syndrome (multi-lineage cytopenias)” (62, 63). Recently Hadjadj et al. reported the findings obtained from genetic studies performed in a cohort of 80 individuals with Evans Syndrome. “In 52 patients (65%) a genetic diagnosis was made. Forty nine patients carried germline mutations while 3 carried somatic variants. Thirty-two patients (40%) had pathogenic mutations in one of 9 genes known to be associated to primary immunodeficiencies (*TNFRSF6, CTLA4, STAT3, PIK3CD, CBL, ADAR1, LRBA, RAG1*, and *KRAS*), whereas 20 patients (25%) carried probable pathogenic variants in 16 genes that had not previously been reported in the context of autoimmune disease. No genetic abnormalities were found in the remaining 28 affected individuals (35%)” (64). The patients in which a genetic diagnosis was achieved, displayed a more severe clinical phenotype and were often refractory to conventional treatment (64). This data underline the importance of pursuing a genetic diagnosis of PID in pediatric Evans syndrome or when cytopenias do not respond to conventional treatment or manifest with a relapsing course (2, 65–68).

Corticosteroids are the first line treatment in AIHA. Around 80% of the patients achieves remission with this therapeutic approach (69). For ITP, the standard of care is represented by corticosteroids together with high-dose intravenous immunoglobulin (IVIG) (70). Rituximab, an anti-CD20 monoclonal antibody, is commonly used in PID when first line treatments fail (71, 72). In a cohort of 25 patients with common variable immune deficiency, treated for refractory cytopenia, Rituximab treatment was more effective than immunosuppressant or immune modulators (73). Rituximab is generally considered an optimal treatment in PID associated blood cytopenias, however relapsing of disease has been observed. Rituximab treatment failure is often linked to the drug ability to deplete only maturing B cells while sparing antibody producing long-lived plasma cells that sustain the autoimmune phenomena. To circumvent this limitation, Bortezomib, a proteasome inhibitor approved for treatment of multiple myeloma that target plasma cells, has been useful in treating refractory cytopenia associated with immune dysregulation (2, 74). Bortezomib has shown efficacy in 4 out of 5 cases of PID with refractory autoimmune cytopenias in the peri transplant period (75). A novel promising therapy that also targets plasma cells and plasmablasts is the anti-CD38 monoclonal antibody, Daratumumab (76). Schuetz et al. reported 3 patients with life-threatening post-transplant AIHA in which daratumumab was effective. In 2 patients the treatment was curative at a follow up of 18 and 13 months after therapy. The third patient had immediate benefit but relapsed 8 months after the drug administration (77). Advancement in understanding the molecular basis of immune dysregulation has allowed the development of many more targeted therapies that may be useful in the treatment of autoimmune cytopenias. Belimumab is a human mAb that inhibits B-cell-activating factor (BAFF). It has been used successfully in the treatment of blood cytopenias in patients with systemic lupus erythematosus (78). Furthermore, anti-A proliferation-inducing ligand (APRIL) antibody is being evaluated for safety and efficacy in the treatment of refractory cytopenias but has not yet been trialed in PID (79). A small molecule inhibitor of Bruton's tyrosine kinase, ibrutinib, has shown efficacy in the treatment of refractory AIHA among chronic lymphocytic leukemia patients (80); eculizumab, an anti-C5, terminal complement inhibitor, has been administered to patient that were unresponsive to other B cell targeted treatment with promising results (81). Anti-CD22 monoclonal antibody, epratuzumab, has also being trialed in refractory cytopenias in patients with SLE and could become another drug to use in PID associated cytopenias (82).

## Precision Medicine in Disorders of Hyperinflammation

### Hemophagocytic Lymphohistiocytosis

Hemophagocytic lymphohistiocytosis (HLH) is a disorder of hyperinflammation that requires aggressive immunosuppression. It was originally named “Familial haemophagocytic reticulosis” in 1952 when it was first described as a genetic disorder in two siblings affected by cytopenias, coagulopathy, and elevated fevers (83). The clinical spectrum of the syndrome and the diagnostic criteria have since evolved. To fit the diagnosis of HLH patients need either a genetic diagnosis of HLH or to meet five out of the eight established clinical criteria for HLH (HLH-2004) (84, 85).

Multiple genes have been associated with the disease and most of them encode for proteins that regulate NK and CTL function or control EBV-driven lymphoproliferation (86–92). However, there are still many cases in which a genetic diagnosis is not achieved.

In 2018 the HLH steering committee of the Histiocyte Society divided HLH in 3 different categories: (1) primary HLH, when HLH associated genetic mutations are identified, (2) MAS-HLH, when there is an underlying autoimmune condition that maybe causative, and (3) secondary HLH, when there is an underlying medical condition (malignancy, infection, metabolic disorder, or primary or secondary immunodeficiencies). They also proposed that, when 2004 HLH criteria are present, the first line treatment should be etoposide-based chemotherapy regimen and afterwards, in selected cases, HSCT (93). Overall survival after HSCT for genetic forms of HLH is still poor (50%). Recently Allen et al. have shown that use of reduced-intensity condition is associated with reduced morbidity and mortality (94). Other treatments have shown to be effective in ameliorating HLH outcomes. Marsh et al. analyzed retrospectively the medical records of 22 pediatric and adult patients treated with alemtuzumab, a monoclonal antibody targeting CD52, for the treatment of refractory HLH. They concluded that this treatment was an effective salvage agent for HLH that led to improved patients survival to HCT (95). Alemtuzumab is currently being evaluated in a clinical trial that enrolles subjects affected by primary HLH (NCT02472054).

In 2018, Chinn et al. reported that in 48 patients with HLH, whole exome sequencing analysis was able to find a genetic diagnosis of primary immune deficiency in 12 patients. These genetic findings offered the opportunity to target the exact molecular mechanism underpinning the disease. As an example, in a patient diagnosed with STAT3 GOF, Jakinib therapy was successful in reversing the HLH clinical and laboratory manifestations (46, 96). A phase I trial evaluating ruxolitinib for patients with HLH is now recruiting (NCT02400463).

*In vitro* and *in vivo* studies demonstrated that IFN-γ is a major driver of inflammation in HLH. On the bases of this observations an international phase II/III clinical trial (NCT01818492) was originally open to allow the use of a monoclonal antibody against IFN- γ, emapalumab, in primary and MAS-HLH (97, 98). In addition, emapalumab has been recently approved by FDA for “treatment of pediatric (new born and older) and adult patients with primary haemophagocytic lymphohistiocytosis (HLH) with refractory, recurrent, or progressive disease or intolerance to conventional HLH therapy.”

### Interferonopathies

The interferonopathies are a group of disorders due to hyperactivation of type I interferon response to multiple triggers, including damaged nucleic acid in the cytosol. DNA sequences are recognized by Cyclic guanosine monophosphate adenosine monophosphate synthase, a sensor molecule that activates the stimulator of interferon genes (STING) (99–101). STING promotes the inflammatory response by activating either interferon regulatory factor 3 or nuclear factor kappa B (NF-κB) (102–104). STING-associated diseases include early onset severe vasculopathy (105) and familial chilblain lupus (106). Both diseases are associated with GOF mutations in *TMEM173*, the gene that encodes for the STING protein. Affected individuals suffer from early onset cold-induced blistering rash, increased inflammatory markers, and fever. Small vessel vasculopathy that can lead to necrosis of digits is a severe but not uncommon complication of the disease (105). Dysregulated type 1 interferon signatures and hyperinflammation have also been associated with genetic defects in the components of the immunoproteasome. This is a complex proteolytic machinery derived from the constitutive proteasome and is expressed in immune cells. The immunoproteasome has key functions in the immune system because it processes proteins for antigen presentation and regulates activation of the NF-κB pathway, and management of oxidative stress (107, 108). Mutations in multiple genes encoding for immunoproteasome subunits, such as PSMA3, PSMB4, PSMB8, and PSMB9, have been linked to autoinflammatory syndromes. In addition, a similar but not completely overlapping phenotype has been associated to *POMP* mutations. This gene encodes for a protein that is crucial for immunoproteasome assembly (107, 109). The clinical phenotype in this group of diseases is summarized by the acronym CANDLE, which stands for early onset Chronic Atypical Neutrophilic Dermatosis, Lipodystrophy, and Elevated temperature. In contrast, *POMP* mutations lead to a distinct phenotype that is characterized by immune deficiency, neonatal-onset Sweet syndrome, and autoimmunity (107).

Multiple immunosuppressant agents including corticosteroids, methotrexate, infliximab, etanercept, 6-mercaptopurine, azathioprine, cyclophosphamide, IL-1 antagonist, and rituximab have been used alone or in combination in patients with CANDLE, but the response has been limited (105, 110, 111). Because STING activation results in upregulation of type I interferon expression and activation of the JAK/STAT pathway, targeted therapy with Jakinibs has been attempted in order to neutralize the dysregulated interferon signature, with good results. In particular, treatment with baricitinib, tofacitinib, and ruxolitinib can decrease the severity of clinical manifestations and inflammatory markers in CANDLE syndrome and STING-associated vasculopathy (105, 106, 110–112). Small-molecule antagonists of STING have been studied in a mouse model with encouraging results and may represent an alternative therapeutic strategy in the future (113). In POMP disease, a more traditional immunosuppressive approach with steroids, rapamycin, and rituximab has been helpful in limiting some of the disease manifestations; however, based on recent findings of increased interferon signature in this disease, a more targeted approach could be considered in the future (107).

### Inflammasome Disorders

The innate immune system is a key player in mounting adequate inflammatory responses against pathogens and molecules resulting from cellular damage. In this regard, rapid availability of proinflammatory cytokines like IL-1 and IL-18 represents a key mechanism of inflammation. Dysregulation in IL-1 production and signaling have been associated with mutations in the *IL1RN* genes and *NLRP3*. These mutations lead within the first few months of life to autoinflammatory diseases, deficiency of IL-1 receptor antagonist and cryopyrin-associated periodic syndromes (CAPS), respectively. NLRP3 is the best studied cytoplasmic sensor molecule of the inflammasome. It is a protein composed of three part that belongs to the NLR family and contains an amino-terminal PYRIN (PYD) domain, a nucleotide-binding NACHT domain, and a carboxy-terminal leucine-rich repeat (LRR) domain. Upon stimulation, the inactive pro-IL-1β and pro-IL-18 molecules are cleaved into their active forms by the proteolytic enzymes activated by NLRP3 inflammasome. Constitutive overactivation of the inflammasome is the result of *NLRP3* mutations in patients affected by CAPS (114). In 2001, *NLRP3* mutations were first described to be associated to familial cold autoinflammatory syndrome (115) and Muckle-Wells syndrome (116). The first one is an early-onset disease with intermittent cold induced neutrophilic urticaria, arthralgia, increased inflammatory markers, and fevers. Muckle-Wells disease also manifests during infancy with mild to moderate fever, rash without itchiness, swollen and painful joints, and in some cases conjunctivitis. During the teenage years patients develop hearing loss caused by progressive damage of the choclear nerve. In about 30% of patients with Muckle-Wells syndrome deposition of amyloid cause progressive kidney disease (116).

In 2002, *NLRP3* mutations were found as the cause of neonatal onset multisystem inflammatory disease (NOMID), also called chronic infantile neurological cutaneous articular (CINCA) syndrome (117, 118). Together with many features present in the other relatively milder forms of CAPS, patients with NOMID often manifest CNS involvement, with presence of cerebral calcifications. Thus, far the literature reports about 200 *NLRP3* mutations that lead to CAPS clinical features (119). These mutations cause spontaneous inflammasome formation despite lack of activating signals and are classified as gain-of-function (117, 118).

**Figure 2:**
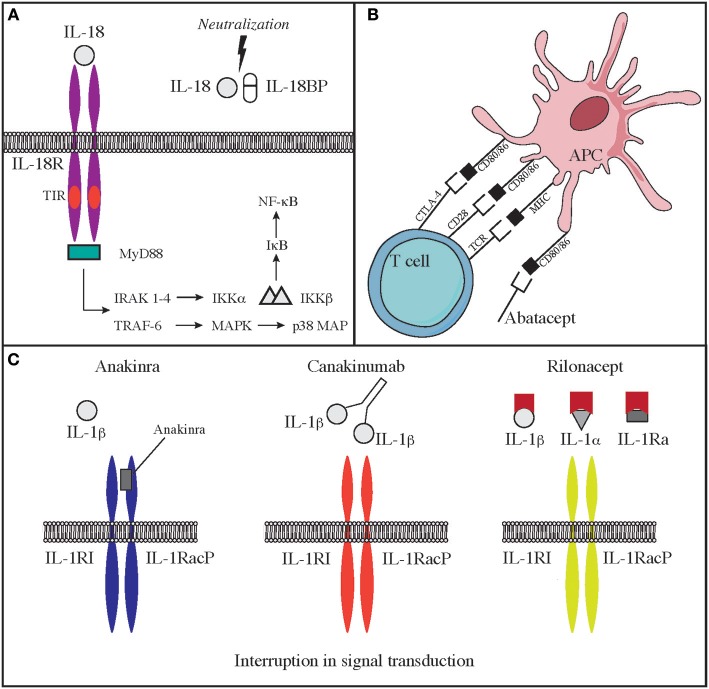
Mechanisms of action of **(A)** IL-18 binding protein **(B)** Abatacept **(C)** IL-1 antagonists.

Deficiency of IL-1 receptor antagonist (DIRA) is due to biallelic loss-of-function mutations of the *IL1RN* gene. These mutations lead to reduced expression of an antagonist of IL-1 signaling and consequent elevated production of IL-1β. The disease has some overlapping features with CAPS. The onset is typically neonatal, and patients suffer from fevers, skin rash, lesions of the oral mucosa, joint swelling, and bone abnormalities, including osteolytic lesions, wide clavicles, and ribs and periosteal elevation along the long bones (37, 120). Use of IL-1 antagonists is efficacious in improving some of the clinical manifestations of severe inflammation in these patients. In particular, the recombinant IL-1 receptor antagonist Anakinra, has a short half-life, binds the IL-1 receptor and impairs the binding of IL-1β and IL-1α. Furthermore, IL-1R1 and IL-1RAcP can be linked to the Fc portion of IgG1 that binds IL-1α and IL-1β to generate a fusion proteined named Rilonacept. Finally, canakinumab is a humanized IgG monoclonal antibody binds IL-1β in a selective manner. Both rilonacept and canakinumab have longer half-life as compared to anakinra (Figure 2C).

Treatment with this newly discovered drugs significantly improves clinical manifestations, inflammatory markers, and frequency of disease episodes in CAPS and DIRA (115, 121–123). Use of anakinra is highly efficacious in the treatment of NOMID. Canakinumab and Rilonacept have been approved by the FDA for the therapy of CAPS.

Another disease that highly benefits from a targeted approach with monoclonal antibodies against interleukins is the autoinflammatory syndrome due to GOF mutations in the *NLRC4* gene (NLR family CARD domain-containing protein 4). This is a very early onset disease that presents with macrophage activation syndrome and enterocolitis (124–126). The activation of NLRC4 protein causes both caspase-1 and caspase-8 activation and consequent overproduction of proinflammatory cytokines. Patients show increased levels in the blood of IL-18 and IL-1β. The latter seems responsible of increased cell death (124, 125, 127, 128). While administration of IL-1 inhibitory molecules alone has been able to only partially modulate the clinical manifestations of the disease, therapy with recombinant IL-18 binding protein (tadekinig-alfa) was able to improve disease manifestations in a subject affected by NLRC4 GOF disease, and a phase 3 clinical trial (NCT03113760) is currently under way (127) (Figure 2A). In addition, a combination of rapamycin and IL-1 inhibition has been attempted with the aim of reducing activation of caspase-1 and consequent overproduction of IL-1 and IL-18. Such multi-drug approach was able to improve clinical manifestations and should be considered in NLRC4 GOF patients especially when IL-18 binding protein is not available (127, 129).

## Gene Therapy

Gene therapy is currently one of the most appealing targeted therapeutic approaches to treat selected inborn errors of immunity. Some well-know PID due to LOS mutations, like Wiskott-Aldrich syndrome and adenosine deaminase deficiency, can be successfully treated trough the replacement of a wild type copy of the mutated gene in autologous HSCs (130). A similar gene therapy approach though would not be effective in reversing the clinical phenotype of immunedysregulation and hyperinflammation disorders due to GOF mutations. In this context, addition, or insertion of a wild type copy of the gene would still leave the defective gene copy intact and able to generate a mutant protein that impairs downstream signaling. GOF diseases are caused by hyperactivation of crucial regulatory pathway of the immune system and gene therapy should aim to decrease the activity of the mutant protein. Possible strategies are: (1) targeted knock down of mutant RNA to reduce the expression of the hyperfunctioning protein responsible for the disease phenotype or (2) gene editing approaches aimed to either correct the pathogenic mutation or disrupt the mutant allele while sparing the wild type allele in the case of autosomal dominant diseases.

## Conclusion

Improved understanding of the molecular mechanisms underpinning the pathogenesis of several newly described PIDs allowed for the development of targeted therapeutic strategies to treat affected patients. This novel mechanism-based approach, called “precision medicine,” by utilizing small molecules and biologics, is effective in reversing clinical manifestations of immunedysregulation and hyperinflammation in many PIDs. In addition, in patients in which HSCT is required, targeted treatments have been successful in decreasing pre-transplant patients disease burden. These drugs might represent a useful bridge therapy to control immune dysregulation and hyperinflammation with the aim of leading patients to transplantation in better clinical status and consequently improving HSCT outcomes.

## Author Contributions

OD analyzed evidence from literature and wrote the manuscript. RC analyzed evidence from literature and helped supervise the project. EC elaborated the table and figure. LN supervised the project and critically reviewed the final draft. All the authors approved the submitted manuscript and agreed to be accountable for the content of the work.

### Conflict of Interest Statement

The authors declare that the research was conducted in the absence of any commercial or financial relationships that could be construed as a potential conflict of interest.

## References

[1] Picard C Bobby Gaspar H Al-Herz W Bousfiha A Casanova JL Chatila T. International union of immunological societies: 2017 primary immunodeficiency diseases committee report on inborn errors of immunity. J Clin Immunol. (2018) 38:96–128. 10.1007/s10875-017-0464-9 29226302 PMC5742601

[2] Delmonte OM Schuetz C Notarangelo LD. RAG deficiency: two genes, many diseases. J Clin Immunol. (2018) 38:646–55. 10.1007/s10875-018-0537-4 30046960 PMC6643099

[3] Krummel MF Allison JP. Pillars article: CD28 and CTLA-4 have opposing effects on the response of T cells to stimulation. Journal of experimental medicine. 1995. 182: 459–465. J Immunol. (2011) 187:3459–65. 10.1084/jem.182.2.459 21934097

[4] Herrero-Beaumont G Martínez Calatrava MJ Castañeda S. Abatacept mechanism of action: concordance with its clinical profile. Reumatol Clin. (2012) 8:78–83. 10.1016/j.reumae.2011.08.004 22104048

[5] Kuehn HS Ouyang W Lo B Deenick EK Niemela JE Avery DT. Immune dysregulation in human subjects with heterozygous germline mutations in CTLA4. Science. (2014) 345:1623–7. 10.1126/science.1255904 25213377 PMC4371526

[6] Schubert D Bode C Kenefeck R Hou TZ Wing JB Kennedy A. Autosomal dominant immune dysregulation syndrome in humans with CTLA4 mutations. Nat Med. (2014) 20:1410–6. 10.1038/nm.3746 25329329 PMC4668597

[7] Schwab C Gabrysch A Olbrich P Patiño V Warnatz K Wolff D. Phenotype, penetrance, and treatment of 133 cytotoxic T-lymphocyte antigen 4-insufficient subjects. J Allergy Clin Immunol. (2018) 142:1932–46. 10.1016/j.jaci.2018.02.055 29729943 PMC6215742

[8] Lee S Moon JS Lee CR Kim HE Baek SM Hwang S. Abatacept alleviates severe autoimmune symptoms in a patient carrying a *de novo* variant in CTLA-4. J Allergy Clin Immunol. (2016) 137:327–30. 10.1016/j.jaci.2015.08.036 26478010

[9] Navarini AA Hruz P Berger CT Hou TZ Schwab C Gabrysch A. Vedolizumab as a successful treatment of CTLA-4-associated autoimmune enterocolitis. J Allergy Clin Immunol. (2017) 139:1043–6.e1045. 10.1016/j.jaci.2016.08.042 27908448

[10] Slatter MA Engelhardt KR Burroughs LM Arkwright PD Nademi Z Skoda-Smith S. Hematopoietic stem cell transplantation for CTLA4 deficiency. J Allergy Clin Immunol. (2016) 138:615–9.e611. 10.1016/j.jaci.2016.01.045 27102614

[11] Lo B Zhang K Lu W Zheng L Zhang Q Kanellopoulou C. Autoimmune disease. Patients with LRBA deficiency show CTLA4 loss and immune dysregulation responsive to abatacept therapy. Science. (2015) 349:436–40. 10.1126/science.aaa1663 26206937

[12] Lopez-Herrera G Tampella G Pan-Hammarström Q Herholz P Trujillo-Vargas CM Phadwal K. Deleterious mutations in LRBA are associated with a syndrome of immune deficiency and autoimmunity. Am J Hum Genet. (2012) 90:986–1001. 10.1016/j.ajhg.2012.04.015 22608502 PMC3370280

[13] Lévy E Stolzenberg MC Bruneau J Breton S Neven B Sauvion S. LRBA deficiency with autoimmunity and early onset chronic erosive polyarthritis. Clin Immunol. (2016) 168:88–93. 10.1016/j.clim.2016.03.006 27057999

[14] Alkhairy OK Abolhassani H Rezaei N Fang M Andersen KK Chavoshzadeh Z. Spectrum of phenotypes associated with mutations in LRBA. J Clin Immunol. (2016) 36:33–45. 10.1007/s10875-015-0224-7 26707784

[15] Bratanič N Kovač J Pohar K Trebušak Podkrajšek K Ihan A Battelino T. Multifocal gastric adenocarcinoma in a patient with LRBA deficiency. Orphanet J Rare Dis. (2017) 12:131. 10.1186/s13023-017-0682-5 28720148 PMC5516372

[16] Gámez-Díaz L August D Stepensky P Revel-Vilk S Seidel MG Noriko M. The extended phenotype of LPS-responsive beige-like anchor protein (LRBA) deficiency. J Allergy Clin Immunol. (2016) 137:223–30. 10.1016/j.jaci.2015.09.025 26768763

[17] Charbonnier LM Janssen E Chou J Ohsumi TK Keles S Hsu JT. Regulatory T-cell deficiency and immune dysregulation, polyendocrinopathy, enteropathy, X-linked-like disorder caused by loss-of-function mutations in LRBA. J Allergy Clin Immunol. (2015) 135:217–27. 10.1016/j.jaci.2014.10.019 25468195 PMC4289093

[18] Revel-Vilk S Fischer U Keller B Nabhani S Gámez-Díaz L Rensing-Ehl A. Autoimmune lymphoproliferative syndrome-like disease in patients with LRBA mutation. Clin Immunol. (2015) 159:84–92. 10.1016/j.clim.2015.04.007 25931386

[19] Seidel MG Hirschmugl T Gamez-Diaz L Schwinger W Serwas N Deutschmann A. Long-term remission after allogeneic hematopoietic stem cell transplantation in LPS-responsive beige-like anchor (LRBA) deficiency. J Allergy Clin Immunol. (2015) 135:1384–90.e1–8. 10.1016/j.jaci.2014.10.048 25539626 PMC4429722

[20] Lucas CL Kuehn HS Zhao F Niemela JE Deenick EK Palendira U. Dominant-activating germline mutations in the gene encoding the PI(3)K catalytic subunit p110δ result in T cell senescence and human immunodeficiency. Nat Immunol. (2014) 15:88–97. 10.1038/ni.2771 24165795 PMC4209962

[21] Angulo I Vadas O Garçon F Banham-Hall E Plagnol V Leahy TR. Phosphoinositide 3-kinase δ gene mutation predisposes to respiratory infection and airway damage. Science. (2013) 342:866–71. 10.1126/science.1243292 24136356 PMC3930011

[22] Lucas CL Zhang Y Venida A Wang Y Hughes J McElwee J. Heterozygous splice mutation in PIK3R1 causes human immunodeficiency with lymphoproliferation due to dominant activation of PI3K. J Exp Med. (2014) 211:2537–47. 10.1084/jem.20141759 25488983 PMC4267241

[23] Olbrich P Lorenz M Cura Daball P Lucena JM Rensing-Ehl A Sanchez B. Activated PI3Kδ syndrome type 2: Two patients, a novel mutation, and review of the literature. Pediatr Allergy Immunol. (2016) 27:640–4. 10.1111/pai.12585 27116393

[24] Petrovski S Parrott RE Roberts JL Huang H Yang J Gorentla B. Dominant splice site mutations in PIK3R1 cause hyper IgM syndrome, lymphadenopathy and short stature. J Clin Immunol. (2016) 36:462–71. 10.1007/s10875-016-0281-6 27076228 PMC5690581

[25] Coulter TI Chandra A Bacon CM Babar J Curtis J Screaton N. Clinical spectrum and features of activated phosphoinositide 3-kinase δ syndrome: a large patient cohort study. J Allergy Clin Immunol. (2017) 139:597–606.e4. 10.1016/j.jaci.2016.06.021 27555459 PMC5292996

[26] Elkaim E Neven B Bruneau J Mitsui-Sekinaka K Stanislas A Heurtier L. Clinical and immunologic phenotype associated with activated phosphoinositide 3-kinase δ syndrome 2: a cohort study. J Allergy Clin Immunol. (2016) 138:210–8.e9. 10.1016/j.jaci.2016.03.022 27221134

[27] Condliffe AM Chandra A. Respiratory manifestations of the activated phosphoinositide 3-kinase delta syndrome. Front Immunol. (2018) 9:338. 10.3389/fimmu.2018.00338 29556229 PMC5844940

[28] Maccari ME Abolhassani H Aghamohammadi A Aiuti A Aleinikova O Bangs C. Disease evolution and response to rapamycin in activated phosphoinositide 3-kinase δ syndrome: the european society for immunodeficiencies-activated phosphoinositide 3-kinase δ syndrome registry. Front Immunol. (2018) 9:543. 10.3389/fimmu.2018.00543 29599784 PMC5863269

[29] Nademi Z Slatter MA Dvorak CC Neven B Fischer A Suarez F. Hematopoietic stem cell transplant in patients with activated PI3K delta syndrome. J Allergy Clin Immunol. (2017) 139:1046–9. 10.1016/j.jaci.2016.09.040 27847301

[30] Okano T Imai K Tsujita Y Mitsuiki N Yoshida K Kamae C. Hematopoietic stem cell transplantation for progressive combined immunodeficiency and lymphoproliferation in patients with activated phosphatidylinositol-3-OH kinase δ syndrome type 1. J Allergy Clin Immunol. (2019) 143:266–75. 10.1016/j.jaci.2018.04.032 29778502

[31] Rao VK Webster S Dalm VASH Šedivá A van Hagen PM Holland S. Effective “activated PI3Kδ syndrome”-targeted therapy with the PI3Kδ inhibitor leniolisib. Blood. (2017) 130:2307–16. 10.1182/blood-2017-08-801191 28972011 PMC5701526

[32] Compagno M Wang Q Pighi C Cheong TC Meng FL Poggio T. Phosphatidylinositol 3-kinase δ blockade increases genomic instability in B cells. Nature. (2017) 542:489–93. 10.1038/nature21406 28199309 PMC5382874

[33] Casanova JL Holland SM Notarangelo LD. Inborn errors of human JAKs and STATs. Immunity. (2012) 36:515–28. 10.1016/j.immuni.2012.03.016 22520845 PMC3334867

[34] O'Shea JJ Plenge R. JAK and STAT signaling molecules in immunoregulation and immune-mediated disease. Immunity. (2012) 36:542–50. 10.1016/j.immuni.2012.03.014 22520847 PMC3499974

[35] Dupuis S Jouanguy E Al-Hajjar S Fieschi C Al-Mohsen IZ Al-Jumaah S. Impaired response to interferon-alpha/beta and lethal viral disease in human STAT1 deficiency. Nat Genet. (2003) 33:388–91. 10.1038/ng1097 12590259

[36] Dupuis S Dargemont C Fieschi C Thomassin N Rosenzweig S Harris J Impairment of mycobacterial but not viral immunity by a germline human STAT1 mutation. Science. (2001) 293:300–3. 10.1126/science.1061154 11452125

[37] Liu L Okada S Kong XF Kreins AY Cypowyj S Abhyankar A. Gain-of-function human STAT1 mutations impair IL-17 immunity and underlie chronic mucocutaneous candidiasis. J Exp Med. (2011) 208:1635–48. 10.1084/jem.20110958 21727188 PMC3149226

[38] van de Veerdonk FL Plantinga TS Hoischen A Smeekens SP Joosten LA Gilissen C. STAT1 mutations in autosomal dominant chronic mucocutaneous candidiasis. N Engl J Med. (2011) 365:54–61. 10.1056/NEJMoa1100102 21714643

[39] Toubiana J Okada S Hiller J Oleastro M Lagos Gomez M Aldave Becerra JC. Heterozygous STAT1 gain-of-function mutations underlie an unexpectedly broad clinical phenotype. Blood. (2016) 127:3154–64. 10.1182/blood-2015-11-679902 27114460 PMC4920021

[40] Kumar N Hanks ME Chandrasekaran P Davis BC Hsu AP Van Wagoner NJ. Gain-of-function signal transducer and activator of transcription 1 (STAT1) mutation-related primary immunodeficiency is associated with disseminated mucormycosis. J Allergy Clin Immunol. (2014) 134:236–9. 10.1016/j.jaci.2014.02.037 24709374 PMC4125455

[41] Sampaio EP Hsu AP Pechacek J Bax HI Dias DL Paulson ML. Signal transducer and activator of transcription 1 (STAT1) gain-of-function mutations and disseminated coccidioidomycosis and histoplasmosis. J Allergy Clin Immunol. (2013) 131:1624–34. 10.1016/j.jaci.2013.01.052 23541320 PMC3746066

[42] Uzel G Sampaio EP Lawrence MG Hsu AP Hackett M Dorsey MJ. Dominant gain-of-function STAT1 mutations in FOXP3 wild-type immune dysregulation-polyendocrinopathy-enteropathy-X-linked-like syndrome. J Allergy Clin Immunol. (2013) 131:1611–23. 10.1016/j.jaci.2012.11.054 23534974 PMC3672257

[43] Depner M Fuchs S Raabe J Frede N Glocker C Doffinger R. The extended clinical phenotype of 26 patients with chronic mucocutaneous candidiasis due to gain-of-function mutations in STAT1. J Clin Immunol. (2016) 36:73–84. 10.1007/s10875-015-0214-9 26604104 PMC4718942

[44] Ma CS Wong N Rao G Avery DT Torpy J Hambridge T. Monogenic mutations differentially affect the quantity and quality of T follicular helper cells in patients with human primary immunodeficiencies. J Allergy Clin Immunol. (2015) 136:993–1006.e1. 10.1016/j.jaci.2015.05.036 26162572 PMC5042203

[45] Leiding JW Okada S Hagin D Abinun M Shcherbina A Balashov DN. Hematopoietic stem cell transplantation in patients with gain-of-function signal transducer and activator of transcription 1 mutations. J Allergy Clin Immunol. (2018) 141:704–17.e5. 10.1016/j.jaci.2017.03.049 28601685 PMC7802430

[46] Forbes LR Vogel TP Cooper MA Castro-Wagner J Schussler E Weinacht KG Jakinibs for the treatment of immune dysregulation in patients with gain-of-function signal transducer and activator of transcription 1 (STAT1) or STAT3 mutations. J Allergy Clin Immunol. (2018) 142:1665–9. 10.1016/j.jaci.2018.07.020 30092289 PMC6322659

[47] Higgins E Al Shehri T McAleer MA Conlon N Feighery C Lilic D. Use of ruxolitinib to successfully treat chronic mucocutaneous candidiasis caused by gain-of-function signal transducer and activator of transcription 1 (STAT1) mutation. J Allergy Clin Immunol. (2015) 135:551–3. 10.1016/j.jaci.2014.12.1867 25662309

[48] Meesilpavikkai K Dik WA Schrijver B Nagtzaam NMA Posthumus-van Sluijs SJ van Hagen PM. Baricitinib treatment in a patient with a gain-of-function mutation in signal transducer and activator of transcription 1 (STAT1). J Allergy Clin Immunol. (2018) 142:328–30.e2. 10.1016/j.jaci.2018.02.045 29729898

[49] Hirahara K Schwartz D Gadina M Kanno Y O'Shea JJ. Targeting cytokine signaling in autoimmunity: back to the future and beyond. Curr Opin Immunol. (2016) 43:89–97. 10.1016/j.coi.2016.10.001 27821272

[50] Roskoski R Jr. Janus kinase (JAK) inhibitors in the treatment of inflammatory and neoplastic diseases. Pharmacol Res. (2016) 111:784–803. 10.1016/j.phrs.2016.07.038 27473820

[51] Vargas-Hernández A Mace EM Zimmerman O Zerbe CS Freeman AF Rosenzweig S. Ruxolitinib partially reverses functional natural killer cell deficiency in patients with signal transducer and activator of transcription 1 (STAT1) gain-of-function mutations. J Allergy Clin Immunol. (2018) 141:2142–55.e5. 10.1016/j.jaci.2017.08.040 29111217 PMC5924437

[52] Weinacht KG Charbonnier LM Alroqi F Plant A Qiao Q Wu H. Ruxolitinib reverses dysregulated T helper cell responses and controls autoimmunity caused by a novel signal transducer and activator of transcription 1 (STAT1) gain-of-function mutation. J Allergy Clin Immunol. (2017) 139:1629–40.e2. 10.1016/j.jaci.2016.11.022 28139313 PMC5482293

[53] Aldave JC Cachay E Núñez L Chunga A Murillo S Cypowyj S. A 1-year-old girl with a gain-of-function STAT1 mutation treated with hematopoietic stem cell transplantation. J Clin Immunol. (2013) 33:1273–5. 10.1007/s10875-013-9947-5 24105462

[54] Flanagan SE Haapaniemi E Russell MA Caswell R Allen HL De Franco E. Activating germline mutations in STAT3 cause early-onset multi-organ autoimmune disease. Nat Genet. (2014) 46:812–4. 10.1038/ng.3040 25038750 PMC4129488

[55] Haapaniemi EM Kaustio M Rajala HL van Adrichem AJ Kainulainen L Glumoff V. Autoimmunity, hypogammaglobulinemia, lymphoproliferation, and mycobacterial disease in patients with activating mutations in STAT3. Blood. (2015) 125:639–48. 10.1182/blood-2014-04-570101 25349174 PMC4304109

[56] Milner JD Brenchley JM Laurence A Freeman AF Hill BJ Elias KM. Impaired T(H)17 cell differentiation in subjects with autosomal dominant hyper-IgE syndrome. Nature. (2008) 452:773–6. 10.1038/nature06764 18337720 PMC2864108

[57] Haddad E. STAT3: too much may be worse than not enough! Blood. (2015) 125:583–4. 10.1182/blood-2014-11-610592 25614633

[58] Milner JD Vogel TP Forbes L Ma CA Stray-Pedersen A Niemela JE. Early-onset lymphoproliferation and autoimmunity caused by germline STAT3 gain-of-function mutations. Blood. (2015) 125:591–9. 10.1182/blood-2014-09-602763 25359994 PMC4304103

[59] Palmer DC Restifo NP. Suppressors of cytokine signaling (SOCS) in T cell differentiation, maturation, and function. Trends Immunol. (2009) 30:592–602. 10.1016/j.it.2009.09.009 19879803 PMC2787651

[60] Camporeale A Poli V. IL-6, IL-17 and STAT3: a holy trinity in auto-immunity? Front Biosci. (2012) 17:2306–26. 10.2741/4054 22652781

[61] Kane A Deenick EK Ma CS Cook MC Uzel G Tangye SG. STAT3 is a central regulator of lymphocyte differentiation and function. Curr Opin Immunol. (2014) 28:49–57. 10.1016/j.coi.2014.01.015 24594518

[62] Teachey DT Manno CS Axsom KM Andrews T Choi JK Greenbaum BH. Unmasking Evans syndrome: T-cell phenotype and apoptotic response reveal autoimmune lymphoproliferative syndrome (ALPS). Blood. (2005) 105:2443–8. 10.1182/blood-2004-09-3542 15542578

[63] Aladjidi N Leverger G Leblanc T Picat MQ Michel G Bertrand Y. New insights into childhood autoimmune hemolytic anemia: a French national observational study of 265 children. Haematologica. (2011) 96:655–63. 10.3324/haematol.2010.036053 21228033 PMC3084911

[64] Hadjadj J Aladjidi N Fernandes H Leverger G Magérus-Chatinet A Mazerolles F. Pediatric Evans syndrome is associated with a high frequency of potentially damaging variants in immune genes. Blood. (2019) 134:9–21. 10.1182/blood-2018-11-887141 30940614

[65] Teachey DT Greiner R Seif A Attiyeh E Bleesing J Choi J. Treatment with sirolimus results in complete responses in patients with autoimmune lymphoproliferative syndrome. Br J Haematol. (2009) 145:101–6. 10.1111/j.1365-2141.2009.07595.x 19208097 PMC2819393

[66] Heitink-Pollé KM Nijsten J Boonacker CW de Haas M Bruin MC. Clinical and laboratory predictors of chronic immune thrombocytopenia in children: a systematic review and meta-analysis. Blood. (2014) 124:3295–307. 10.1182/blood-2014-04-570127 25305206

[67] Miano M. How i manage evans syndrome and AIHA cases in children. Br J Haematol. (2016) 172:524–34. 10.1111/bjh.13866 26625877

[68] Farruggia P Fioredda F Puccio G Porretti L Lanza T Ramenghi U. Autoimmune neutropenia of infancy: data from the Italian neutropenia registry. Am J Hematol. (2015) 90:E221–2. 10.1002/ajh.24187 26361081

[69] Lechner K Jager U. How I treat autoimmune hemolytic anemias in adults. Blood. (2010) 116:1831–8. 10.1182/blood-2010-03-259325 20548093

[70] Neunert C Lim W Crowther M Cohen A Solberg L Crowther MA. The american society of hematology 2011 evidence-based practice guideline for immune thrombocytopenia. Blood. (2011) 117:4190–207. 10.1182/blood-2010-08-302984 21325604

[71] Wakim M Shah A Arndt PA Garratty G Weinberg K Hofstra T. Successful anti-CD20 monoclonal antibody treatment of severe autoimmune hemolytic anemia due to warm reactive IgM autoantibody in a child with common variable immunodeficiency. Am J Hematol. (2004) 76:152–5. 10.1002/ajh.20072 15164382

[72] Farmer JR Foldvari Z Ujhazi B De Ravin SS Chen K Bleesing JJH. Outcomes and treatment strategies for autoimmunity and hyperinflammation in patients with RAG deficiency. J Allergy Clin Immunol Pract. (2019) 7:1970–85. 10.1016/j.jaip.2019.02.038 30877075 PMC6612449

[73] Gobert D Bussel JB Cunningham-Rundles C Galicier L Dechartres A Berezne A. Efficacy and safety of rituximab in common variable immunodeficiency-associated immune cytopenias: a retrospective multicentre study on 33 patients. Br J Haematol. (2011) 155:498–508. 10.1111/j.1365-2141.2011.08880.x 21981575 PMC3428031

[74] Raedler L. Velcade (Bortezomib) receives 2 New FDA indications: for retreatment of patients with multiple myeloma and for first-line treatment of patients with mantle-cell lymphoma. Am Health Drug Benefits. (2015) 8:135–40. 26629279 PMC4665054

[75] Khandelwal P Davies SM Grimley MS Jordan MB Curtis BR Jodele S. Bortezomib for refractory autoimmunity in pediatrics. Biol Blood Marrow Transplant. (2014) 20:1654–9. 10.1016/j.bbmt.2014.06.032 24979732

[76] van de Donk NW Janmaat ML Mutis T Lammerts van Bueren JJ Ahmadi T Sasser AK. Monoclonal antibodies targeting CD38 in hematological malignancies and beyond. Immunol Rev. (2016) 270:95–112. 10.1111/imr.12389 26864107 PMC4755228

[77] Schuetz C Hoenig M Moshous D Weinstock C Castelle M Bendavid M. Daratumumab in life-threatening autoimmune hemolytic anemia following hematopoietic stem cell transplantation. Blood Adv. (2018) 2:2550–3. 10.1182/bloodadvances.2018020883 30291113 PMC6177653

[78] Furer V Zisman D Pokroy-Shapira E Molad Y Elkayam O Paran D. Systemic lupus erythematosus exacerbation following cessation of belimumab treatment. Scand J Rheumatol. (2016) 45:103–6. 10.3109/03009742.2015.1074277 26515057

[79] Gao Q Li Q Xue Z Wu P Yang X. *In vitro* and *in vivo* evaluation of a humanized anti-APRIL antibody. Curr Mol Med. (2013) 13:464–5. 10.2174/156652413805076867 23331019

[80] Hampel PJ Larson MC Kabat B Call TG Ding W Kenderian SS. Autoimmune cytopenias in patients with chronic lymphocytic leukaemia treated with ibrutinib in routine clinical practice at an academic medical centre. Br J Haematol. (2018) 183:421–7. 10.1111/bjh.15545 30117139 PMC6234062

[81] Ma K Caplan S. Refractory IgG warm autoimmune hemolytic anemia treated with eculizumab: a novel application of anticomplement therapy. Case Rep Hematol. (2016) 2016:9181698. 10.1155/2016/9181698 27092282 PMC4820603

[82] Wallace DJ Hobbs K Clowse ME Petri M Strand V Pike M. Long-term safety and efficacy of epratuzumab in the treatment of moderate-to- severe systemic lupus erythematosus: results from an open-label extension study. Arthrit Care Res. (2016) 68:534–43. 10.1002/acr.22694 26316325

[83] Farquhar JW Claireaux AE. Familial haemophagocytic reticulosis. Arch Dis Child. (1952) 27:519–25. 10.1136/adc.27.136.519 13008468 PMC1988563

[84] Henter JI Horne A Aricó M Egeler RM Filipovich AH Imashuku S. HLH-2004: diagnostic and therapeutic guidelines for hemophagocytic lymphohistiocytosis. Pediatr Blood Cancer. (2007) 48:124–31. 10.1002/pbc.21039 16937360

[85] Jordan MB Allen CE Weitzman S Filipovich AH McClain KL. How I treat hemophagocytic lymphohistiocytosis. Blood. (2011) 118:4041–52. 10.1182/blood-2011-03-278127 21828139 PMC3204727

[86] Pagel J Beutel K Lehmberg K Koch F Maul-Pavicic A Rohlfs AK. Distinct mutations in STXBP2 are associated with variable clinical presentations in patients with familial hemophagocytic lymphohistiocytosis type 5 (FHL5). Blood. (2012) 119:6016–24. 10.1182/blood-2011-12-398958 22451424

[87] Zur Stadt U Beutel K Kolberg S Schneppenheim R Kabisch H Janka G. Mutation spectrum in children with primary hemophagocytic lymphohistiocytosis: molecular and functional analyses of PRF1, UNC13D, STX11, and RAB27A. Hum Mutat. (2006) 27:62–8. 10.1002/humu.20274 16278825

[88] Trizzino A zur Stadt U Ueda I Risma K Janka G Ishii E. Genotype-phenotype study of familial haemophagocytic lymphohistiocytosis due to perforin mutations. J Med Genet. (2008) 45:15–21. 10.1136/jmg.2007.052670 17873118

[89] Sieni E Cetica V Santoro A Beutel K Mastrodicasa E Meeths M. Genotype-phenotype study of familial haemophagocytic lymphohistiocytosis type 3. J Med Genet. (2011) 48:343–52. 10.1136/jmg.2010.085456 21248318 PMC4115201

[90] Zhang K Jordan MB Marsh RA Johnson JA Kissell D Meller J. Hypomorphic mutations in PRF1, MUNC13–4, and STXBP2 are associated with adult-onset familial HLH. Blood. (2011) 118:5794–8. 10.1182/blood-2011-07-370148 21881043 PMC3228496

[91] Qian Y Johnson JA Connor JA Valencia CA Barasa N Schubert J. The 253-kb inversion and deep intronic mutations in UNC13D are present in North American patients with familial hemophagocytic lymphohistiocytosis 3. Pediatr Blood Cancer. (2014) 61:1034–40. 10.1002/pbc.24955 24470399

[92] Zhang K Chandrakasan S Chapman H Valencia CA Husami A Kissell D. Synergistic defects of different molecules in the cytotoxic pathway lead to clinical familial hemophagocytic lymphohistiocytosis. Blood. (2014) 124:1331–4. 10.1182/blood-2014-05-573105 24916509 PMC4141517

[93] Ehl S Astigarraga I von Bahr Greenwood T Hines M Horne A Ishii E. Recommendations for the use of etoposide-based therapy and bone marrow transplantation for the treatment of HLH: consensus statements by the HLH steering committee of the histiocyte society. J Allergy Clin Immunol Pract. (2018) 6:1508–17. 10.1016/j.jaip.2018.05.031 30201097

[94] Allen CE Marsh R Dawson P Bollard CM Shenoy S Roehrs P. Reduced-intensity conditioning for hematopoietic cell transplant for HLH and primary immune deficiencies. Blood. (2018) 132:1438–51. 10.1182/blood-2018-01-828277 29997222 PMC6161764

[95] Marsh RA Allen CE McClain KL Weinstein JL Kanter J Skiles J. Salvage therapy of refractory hemophagocytic lymphohistiocytosis with alemtuzumab. Pediatr Blood Cancer. (2013) 60:101–9. 10.1002/pbc.24188 22522603 PMC3410971

[96] Chinn IK Eckstein OS Peckham-Gregory EC Goldberg BR Forbes LR Nicholas SK. Genetic and mechanistic diversity in pediatric hemophagocytic lymphohistiocytosis. Blood. (2018) 132:89–100. 10.1182/blood-2017-11-814244 29632024 PMC6034641

[97] Henter JI Elinder G Söder O Hansson M Andersson B Andersson U. Hypercytokinemia in familial hemophagocytic lymphohistiocytosis. Blood. (1991) 78:2918–22. 1954380

[98] Jordan MB Hildeman D Kappler J Marrack P. An animal model of hemophagocytic lymphohistiocytosis (HLH): CD8+ T cells and interferon gamma are essential for the disorder. Blood. (2004) 104:735–43. 10.1182/blood-2003-10-3413 15069016

[99] Burdette DL Vance RE. STING and the innate immune response to nucleic acids in the cytosol. Nat Immunol. (2013) 14:19–26. 10.1038/ni.2491 23238760

[100] Diner EJ Burdette DL Wilson SC Monroe KM Kellenberger CA Hyodo M. The innate immune DNA sensor cGAS produces a noncanonical cyclic dinucleotide that activates human STING. Cell Rep. (2013) 3:1355–61. 10.1016/j.celrep.2013.05.009 23707065 PMC3706192

[101] Mackenzie KJ Carroll P Lettice L Tarnauskaite Ž Reddy K Dix F. Ribonuclease H2 mutations induce a cGAS/STING-dependent innate immune response. EMBO J. (2016) 35:831–44. 10.15252/embj.201593339 26903602 PMC4855687

[102] Abe T Barber GN. Cytosolic-DNA-mediated, STING-dependent proinflammatory gene induction necessitates canonical NF-kappaB activation through TBK1. J Virol. (2014) 88:5328–41. 10.1128/JVI.00037-14 24600004 PMC4019140

[103] Takaoka A Taniguchi T. Cytosolic DNA recognition for triggering innate immune responses. Adv Drug Deliv Rev. (2008) 60:847–57. 10.1016/j.addr.2007.12.002 18280611

[104] Takaoka A Wang Z Choi MK Yanai H Negishi H Ban T. DAI (DLM-1/ZBP1) is a cytosolic DNA sensor and an activator of innate immune response. Nature. (2007) 448:501–5. 10.1038/nature06013 17618271

[105] Liu Y Jesus AA Marrero B Yang D Ramsey SE Sanchez GAM. Activated STING in a vascular and pulmonary syndrome. N Engl J Med. (2014) 371:507–18. 10.1056/NEJMoa1312625 25029335 PMC4174543

[106] König N Fiehn C Wolf C Schuster M Cura Costa E Tüngler V. Familial chilblain lupus due to a gain-of-function mutation in STING. Ann Rheum Dis. (2017) 76:468–72. 10.1136/annrheumdis-2016-209841 27566796

[107] Poli MC Ebstein F Nicholas SK de Guzman MM Forbes LR Chinn IK. Heterozygous truncating variants in POMP escape nonsense-mediated decay and cause a unique immune dysregulatory syndrome. Am J Hum Genet. (2018) 102:1126–42. 10.1016/j.ajhg.2018.04.010 29805043 PMC5992134

[108] Budenholzer L Cheng CL Li Y Hochstrasser M. Proteasome structure and assembly. J Mol Biol. (2017) 429:3500–24. 10.1016/j.jmb.2017.05.027 28583440 PMC5675778

[109] Manthiram K Zhou Q Aksentijevich I Kastner DL The monogenic autoinflammatory diseases define new pathways in human innate immunity and inflammation. Nat Immunol. (2017) 18:832–42. 10.1038/ni.3777 28722725

[110] Kim H Sanchez GA Goldbach-Mansky R. Insights from mendelian interferonopathies: comparison of CANDLE, SAVI with AGS, Monogenic Lupus. J Mol Med. (2016) 94:1111–27. 10.1007/s00109-016-1465-5 27678529 PMC5094849

[111] Liu Y Ramot Y Torrelo A Paller AS Si N Babay S. Mutations in proteasome subunit β type 8 cause chronic atypical neutrophilic dermatosis with lipodystrophy and elevated temperature with evidence of genetic and phenotypic heterogeneity. Arthritis Rheum. (2012) 64:895–907. 10.1002/art.33368 21953331 PMC3278554

[112] Sanchez GAM Reinhardt A Ramsey S Wittkowski H Hashkes PJ Berkun Y. JAK1/2 inhibition with baricitinib in the treatment of autoinflammatory interferonopathies. J Clin Invest. (2018) 128:3041–52. 10.1172/JCI98814 29649002 PMC6026004

[113] Haag SM Gulen MF Reymond L Gibelin A Abrami L Decout A. Targeting STING with covalent small-molecule inhibitors. Nature. (2018) 559:269–73. 10.1038/s41586-018-0287-8 29973723

[114] Martinon F Burns K Tschopp J. The inflammasome: a molecular platform triggering activation of inflammatory caspases and processing of proIL-beta. Mol Cell. (2002) 10:417–26. 10.1016/S1097-2765(02)00599-3 12191486

[115] Hoffman HM Mueller JL Broide DH Wanderer AA Kolodner RD. Mutation of a new gene encoding a putative pyrin-like protein causes familial cold autoinflammatory syndrome and Muckle-Wells syndrome. Nat Genet. (2001) 29:301–5. 10.1038/ng756 11687797 PMC4322000

[116] Cuisset L Drenth JP Berthelot JM Meyrier A Vaudour G Watts RA. Genetic linkage of the Muckle-Wells syndrome to chromosome 1q44. Am J Hum Genet. (1999) 65:1054–9. 10.1086/302589 10486324 PMC1288238

[117] Broderick L De Nardo D Franklin BS Hoffman HM Latz E. The inflammasomes and autoinflammatory syndromes. Annu Rev Pathol. (2015) 10:395–424. 10.1146/annurev-pathol-012414-040431 25423351

[118] Feldmann J Prieur AM Quartier P Berquin P Certain S Cortis E. Chronic infantile neurological cutaneous and articular syndrome is caused by mutations in CIAS1, a gene highly expressed in polymorphonuclear cells and chondrocytes. Am J Hum Genet. (2002) 71:198–203. 10.1086/341357 12032915 PMC384980

[119] Sarrauste de Menthière C Terrière S Pugnère D Ruiz M Demaille J Touitou I. INFEVERS: the registry for FMF and hereditary inflammatory disorders mutations. Nucleic Acids Res. (2003) 31:282–5. 10.1093/nar/gkg031 12520003 PMC165478

[120] Aksentijevich I Masters SL Ferguson PJ Dancey P Frenkel J van Royen-Kerkhoff A. An autoinflammatory disease with deficiency of the interleukin-1-receptor antagonist. N Engl J Med. (2009) 360:2426–37. 10.1056/NEJMoa0807865 19494218 PMC2876877

[121] Goldbach-Mansky R Dailey NJ Canna SW Gelabert A Jones J Rubin BI. Neonatal-onset multisystem inflammatory disease responsive to interleukin-1beta inhibition. N Engl J Med. (2006) 355:581–92. 10.1056/NEJMoa055137 16899778 PMC4178954

[122] Hoffman HM Rosengren S Boyle DL Cho JY Nayar J Mueller JL. Prevention of cold-associated acute inflammation in familial cold autoinflammatory syndrome by interleukin-1 receptor antagonist. Lancet. (2004) 364:1779–85. 10.1016/S0140-6736(04)17401-1 15541451 PMC4321997

[123] Hoffman HM Throne ML Amar NJ Sebai M Kivitz AJ Kavanaugh A. Efficacy and safety of rilonacept (interleukin-1 Trap) in patients with cryopyrin-associated periodic syndromes: results from two sequential placebo-controlled studies. Arthritis Rheum. (2008) 58:2443–52. 10.1002/art.23687 18668535

[124] Romberg N Al Moussawi K Nelson-Williams C Stiegler AL Loring E Choi M. Mutation of NLRC4 causes a syndrome of enterocolitis and autoinflammation. Nat Genet. (2014) 46:1135–9. 10.1038/ng.3066 25217960 PMC4177367

[125] Romberg N Vogel TP Canna SW. NLRC4 inflammasomopathies. Curr Opin Allergy Clin Immunol. (2017) 17:398–404. 10.1097/ACI.0000000000000396 28957823 PMC6070355

[126] Canna SW de Jesus AA Gouni S Brooks SR Marrero B Liu Y. An activating NLRC4 inflammasome mutation causes autoinflammation with recurrent macrophage activation syndrome. Nat Genet. (2014) 46:1140–6. 10.1038/ng.3089 25217959 PMC4177369

[127] Canna SW Girard C Malle L de Jesus A Romberg N Kelsen J. Life-threatening NLRC4-associated hyperinflammation successfully treated with IL-18 inhibition. J Allergy Clin Immunol. (2017) 139:1698–701. 10.1016/j.jaci.2016.10.022 27876626 PMC5846100

[128] Rauch I Deets KA Ji DX von Moltke J Tenthorey JL Lee AY. NAIP-NLRC4 inflammasomes coordinate intestinal epithelial cell expulsion with eicosanoid and IL-18 release via activation of caspase-1 and−8. Immunity. (2017) 46:649–59. 10.1016/j.immuni.2017.03.016 28410991 PMC5476318

[129] Barsalou J Blincoe A Fernandez I Dal-Soglio D Marchitto L Selleri S. Rapamycin as an adjunctive therapy for NLRC4 associated macrophage activation syndrome. Front Immunol. (2018) 9:2162. 10.3389/fimmu.2018.02162 30319625 PMC6166634

[130] Fischer A Hacein-Bey Abina S Touzot F Cavazzana M. Gene therapy for primary immunodeficiencies. Clin Genet. (2015) 88:507–15. 10.1111/cge.12576 25708106

